# HLA antibody repertoire in infants suggests selectivity in transplacental crossing

**DOI:** 10.1111/aji.13264

**Published:** 2020-06-16

**Authors:** Dana M. Savulescu, Michelle Groome, Susan C. K. Malfeld, Shabir Madhi, Anthonet Koen, Stephanie Jones, Vania Duxbury, Karine Scheuermaier, Debbie De Assis Rosa, Melinda Suchard

**Affiliations:** ^1^ Centre for Vaccines and Immunology (CVI) National Institute for Communicable Diseases (NICD) A Division of the National Health Laboratory Service Johannesburg Gauteng South Africa; ^2^ Medical Research Council: Respiratory and Meningeal Pathogens Research Unit Faculty of Health Sciences University of the Witwatersrand Johannesburg Gauteng South Africa; ^3^ Department of Science and Technology/National Research Foundation: Vaccine Preventable Diseases Faculty of Health Sciences University of the Witwatersrand Johannesburg Gauteng South Africa; ^4^ Brain Function Research Group School of Physiology Faculty of Health Sciences University of the Witwatersrand Johannesburg Gauteng South Africa; ^5^ School of Molecular and Cell Biology Faculty of Science University of the Witwatersrand Johannesburg Gauteng South Africa; ^6^ Department of Chemical Pathology Faculty of Health Sciences University of the Witwatersrand Johannesburg Gauteng South Africa

**Keywords:** alloimmunity, HLA, HLA antibodies, immune tolerance, placenta, pregnancy, transplacental crossing

## Abstract

**Problem:**

Late in pregnancy, women produce and transfer high amounts of antibodies to the foetus. During gestation, women produce antibodies against human leukocyte antigens (HLA), including antibodies directed at foetal HLA. There is paucity of data on transplacental crossing, specificity and role of HLA antibodies in pregnancy and new‐borns.

**Method of study:**

Using highly sensitive Luminex technology, we measured prevalence of IgG HLA antibodies in 30 mother‐infant pairs six weeks post‐partum. Additionally, in six pregnant women, we measured HLA antibodies longitudinally and HLA‐typed infant DNA to assess whether maternal HLA antibodies were directed at infant specificities.

**Results:**

Overall, 68% of mothers and 44% of infants expressed HLA‐I antibodies and 56% of mothers and 52% of infants expressed HLA‐II antibodies. Infants shared up to 78% of antibodies with their mothers, suggesting that the remaining antibodies were self‐made. Less than 25% of maternal HLA antibodies were detected in infants, possibly due to selection in transplacental crossing. We detected complement‐fixing HLA antibodies in mothers and at low levels in infants. In a third of our pregnant subjects, we detected infant‐directed HLA antibodies.

**Conclusion:**

Our findings raise the possibility of selection in transplacental crossing of HLA antibodies. As HLA antibodies may act as autoantibodies in the neonate, the mechanism of a selective transfer may give important insights into immune tolerance. Findings also suggest that infants start producing their own HLA antibodies in the first weeks of life, which, together with maternally derived antibodies may impact the infant's immune reaction to HLA proteins.

## 1 INTRODUCTION

Human leukocyte antigens (HLA) are proteins responsible for presentation of short peptides to responding T lymphocytes.^1^, ^2^, ^3^ By binding to T‐cell receptors on the surface of T lymphocytes, HLA proteins lead to T cell activation, as well as to recruitment of additional immune players.^2^, ^3^ HLA class I proteins, encoded by the loci HLA‐A, HLA‐B and HLA‐C, are expressed on the surface of all nucleated cells, whereas HLA class II proteins, encoded by the loci DRB1, DQA/B, DRB3,4,5 and DPA/B, are expressed only on the surface of professional antigen‐presenting cells. Together, the seven HLA‐encoding loci are the most polymorphic genes in the human genome, with thousands of alleles expressed in the population.^1^, ^2^, ^3^ This high polymorphism may result in HLA‐induced alloimmunity mediated by antibodies targeted against HLA, referred to as HLA antibodies.

For a long time, HLA antibodies were believed to be expressed only following sensitizing events such as blood transfusions, multiple pregnancies or intravenous drug use. However, modern bead‐based technology has led to the discovery of previously undetectable low levels of HLA antibodies in up to 77 per cent of healthy individuals with no history of sensitizing events.^4^, ^5^, ^6^, ^7^, ^8^, ^9^, ^10^ Such antibodies may be against one or more HLA specificities,^6^, ^11^ although whether they are produced in response to these HLA antigens or just cross‐react with them is not clear at this point.

During pregnancy, women produce and transfer a large quantity of antibodies to the foetus,^12^, ^13^ which is well described for antibodies against pathogens such as measles or pneumococcus.^14^, ^15^, ^16^ This process begins at around 13 weeks of gestation and increases with gestational age, with the majority of antibodies crossing the placenta during the third trimester.^12^, ^17^, ^18^, ^19^, ^20^ Transplacental crossing is mediated by neonatal FcRn receptors, and most antibodies transferred are IgG.^12^, ^20^, ^21^, ^22^, ^23^, ^24^ It has been found that infants born preterm have markedly lower IgG levels at birth compared with term infants.^12^, ^25^ IgA antibodies have also been shown to cross the placenta^13^, ^26^, ^27^; however, this occurs at low levels, as the main mode of transferring IgA antibodies to the baby is through breastfeeding.^28^

Transplacental crossing of antibodies has recently been shown to involve some degree of selection based on post‐translational modifications in the fragment crystalline (Fc) region.^29^, ^30^ In these studies, IgG molecules with digalactosylated glycans in the Fc region were shown to have higher binding affinity to FcRn, leading to more efficient transplacental crossing. Several studies have suggested that post‐translational modifications in Fc regions of antibodies may be antigen‐dependent.^31^, ^32^ However, since no evidence has been found so far for an antigen‐dependent selection in transplacental crossing, we hypothesize that antibodies produced during pregnancy cross the placenta without depending on their target antigen. Intriguingly, in the case of HLA antibodies, placental transfer may result in the presence of maternally derived, paternally directed antibodies in the foetus, potentially acting as autoantibodies. The impact of such antibodies on the foetus and later, infant, is not known.

The production of HLA antibodies during pregnancy was first observed by Van Rood et al in 1958.^33^ Since then, efforts to measure their prevalence and uncover their role have been relatively scarce. It has been shown that HLA antibodies—both non‐specific, and those targeting paternal HLA proteins—develop in many, but not all healthy pregnancies after 20‐28 weeks of gestation.^19^, ^34^, ^35^, ^36^, ^37^ The frequency of these antibodies increases with parity, reaching 74% in women who have had more than two deliveries.^38^ Interestingly, HLA antibodies that appear during pregnancy are not always detected in women several years after delivery but may persist in immunological memory.^39^

The impact of HLA antibodies on pregnancy outcome is not understood. Some studies have found an association between high levels of HLA antibodies in pregnancy, both in early^35^, ^40^ and late^41^, ^42^, ^43^, ^44^ gestational age, and a poor pregnancy outcome. However, other studies suggest that high levels of HLA antibodies in pregnancy have a positive impact on pregnancy outcome.^36^, ^45^ In line with the latter, immunotherapy with paternal lymphocytes resulting in HLA antibody production may be a therapeutic option in women with recurrent miscarriages.^46^, ^47^ Yet a third line of studies, however, has found no substantial correlation between HLA antibodies and pregnancy outcome.^48^, ^49^ Supporting this, is the observation that pregnancy following oocyte donation—associated with a higher risk of developing child‐specific HLA antibodies—is not associated with immunological complications.^50^

The inconsistency arising from existing studies on HLA antibodies in pregnancy demonstrates gaps in our understanding. Similarly, we know little about the development of HLA antibodies in the foetus and after birth. In humans, the foetus has been shown to develop IgM and IgA antibodies.^20^, ^51^, ^52^ IgM is usually the first class of antibody to be produced by B lymphocytes, having high avidity and agglutinating activity, but reduced specificity and antigen affinity.^36^ When the human body further encounters antigen, B lymphocytes switch their isotype from IgM to IgG, which, in addition to being more specific, also possesses the widest range of effector functions.^36^ Based on evidence so far,^12^, ^51^, ^53^ foetuses do not produce their own IgG antibodies, suggesting that IgG—including potential HLA antibodies—present at birth originate in the mothers. The specific time point and triggers for production of IgG in new‐borns are not known.

In the current study, we investigated the HLA antibody profile in women and their infants six weeks post‐partum, as well as in pregnant women. We used two cohorts; a first cohort of six‐week‐old infants and their mothers (referred to as the “mother‐infant cohort”), and a second cohort of pregnant women at different time points in pregnancy and post‐partum (referred to as the “pregnancy cohort”). We detected IgG HLA antibodies and characterized their specificities, sub‐class distribution and expression levels; additionally, we tested the mother‐infant cohort samples for the presence of complement‐fixing HLA antibodies.

## 2 METHODS

### 2.1 Study design and sample collection

#### 2.1.1 Mother‐infant study

Thirty healthy mother‐infant pairs available from a previously completed study on rotavirus vaccine immunogenicity (NCT02215226^54^) were used. The study, conducted in Soweto, South Africa in 2009‐2010, explored the effect of at least an hour abstention from breastfeeding at immunization visits on immune responses of infants to rotavirus vaccine. The study enrolled black South African mother‐infant pairs who were human immunodeficiency virus (HIV)‐negative, at their six weeks post‐partum visit. Blood samples were drawn from all participants at enrolment, immediately before administration of the rotavirus vaccine to the infants. The serum fraction was separated by centrifugation and stored at −70°C until further use. Infant DNA was not available from this cohort.

#### 2.1.2 Longitudinal Pregnancy study

Twenty‐nine women in their first trimester of pregnancy were recruited from collaborating gynaecologist practices (the Charlotte Maxeke Johannesburg Academic Hospital and Netcare private clinics in South Africa). Recruited patients were of both black and white South African ethnicity. All patients were HIV‐negative, multigravida and over 18 years of age at enrolment. Blood samples were drawn from participants at routine visits to the clinic, including during the first (up to 13 weeks of pregnancy), second (14‐26 weeks of pregnancy) and third (27‐40 weeks of pregnancy) trimesters, and/or 8‐11 weeks post‐partum. Buccal swabs were collected from infants at the post‐partum visit and later processed for DNA extraction. Mothers’ serum was stored at −70°C until further use. Samples of six participants with healthy and uncomplicated pregnancies, who had at least two visits to the clinic and for whom buccal swabs were successfully collected from their babies, were analysed and their results presented here as case studies. The remaining participants are not presented here due to missing data such as incomplete infant HLA typing results or maternal serum time points. We did not collect serum from infants in this study.

### 2.2 Measurement of HLA antibody levels

The prevalence of IgG HLA antibodies was measured in serum of mothers and infants from the mother‐infant cohort, as well as that of mothers from the pregnancy cohort. Presence, strength (expression levels) and specificity of antibodies were measured using Luminex technology. Commercial kits for detection of IgG antibodies against HLA class I and HLA class II (LSA class I/II single antigen kits; Immucor, Norcross, Georgia, USA) were used according to manufacturer's instructions. Antibodies detected included anti‐HLA‐A, HLA‐B and HLA‐C for class I, and anti‐DRB1, DRB3,4,5, DQA/B and DPA/B for class II. The data obtained from these experiments were subsequently analysed using Match‐It software (Immucor, Norcross, Georgia, USA), according to manufacturer's instructions. The HLA antibody strength was recorded as adjusted mean fluorescent intensity (AD‐MFI), with additional interpretive comments: positive, weak, possible or absent. Positive and weak were considered positive results, while possible and absent were considered negative results. Highly reactive sera samples were purified by adsorption of non‐specific antibodies using commercial “Sera clean” kit (Immucor), followed by a second analysis of HLA antibodies. For the mother‐infant study, antibody specificities were also labelled as shared (detected in both mother and infant) or mother‐only/infant‐only. For the pregnancy study, HLA antibodies were categorized as baby‐directed (targeting HLA types of the baby) or non‐specific. Additionally, antibodies were categorized as recurring (expressed at more than one time point) or single time point, in order to describe development of new antibodies at various time points.

Complement‐fixing HLA antibodies were detected in serum of 20 mothers and infants from the mother‐infant study by running HLA‐I/II antibody assays as described above but using complement component C3d as a detection agent to bind to complement‐fixing antibodies in serum. After a wash step, bound C3d was detected with fluorescently labelled antibody against C3d (C3d kit, Immucor). The data obtained were subsequently analysed using Match‐It software (Immucor), as described above.

### 2.3 DNA extraction

Buccal swabs of infants in the pregnancy study were soaked in DNA extracting solution (QuickExtract^TM^ DNA Extraction Solution, Epicentre, Madison, WI, USA), followed by DNA extraction using a commercial kit (QuickExtract^TM^ DNA Extraction kit, Epicentre, Madison, WI, USA). Next, DNA was precipitated as follows. Briefly, samples were mixed with sodium acetate in a 1:10 volume, followed by addition of 2.5 volumes of absolute ethanol and incubation at −70°C. Following incubation, the samples were centrifuged, the pellet was washed with 70% ethanol and then eluted with a commercial elution buffer (QIAamp DNA Mini kit, Qiagen, Germany), quantified and stored at −20°C. DNA was not available in the mother‐infant cohort, and therefore no DNA analysis was carried out on these samples.

### 2.4 HLA typing

Class I and II HLA genotyping was performed for DNA prepared from infant samples collected in the pregnancy cohort. Commercial kits for HLA‐A, HLA‐B, HLA‐C, DRB1, DRB3,4,5, DQB1 and DPA/B loci (Immucor, Norcross, Georgia, USA) were used for locus‐specific PCR amplification according to manufacturer's instructions, followed by detection via allele‐specific probes using a Luminex instrument (Bioplex, Biorad) and Match‐It DNA software (Immucor). The typing was reported at the 2/4 ‐ digit resolution.

### 2.5 Statistical analysis

Data analysis was performed using version 8.2.1 (441) of the GraphPad Prism (GraphPad software Inc, San Diego, CA, USA). A *P* value of .05 or lower was considered statistically significant. Variables were compared using ꭕ2 test (for number of specificities per individual) or Mann‐Whitney test (for antibody strength).

### 2.6 Ethics

Our mother‐infant study, conducted on samples from the previously published NCT02215226 study, was approved by the Human Ethics Research Committee of the University of the Witwatersrand with consent for storage and testing (M1611144). The pregnancy study was approved by the Human Ethics Research Committee of the University of the Witwatersrand (M130230).

## 3 RESULTS

### 3.1 Mother‐infant study

#### 3.1.1 HLA antibody specificities in mother and infants

**Figure 1:**
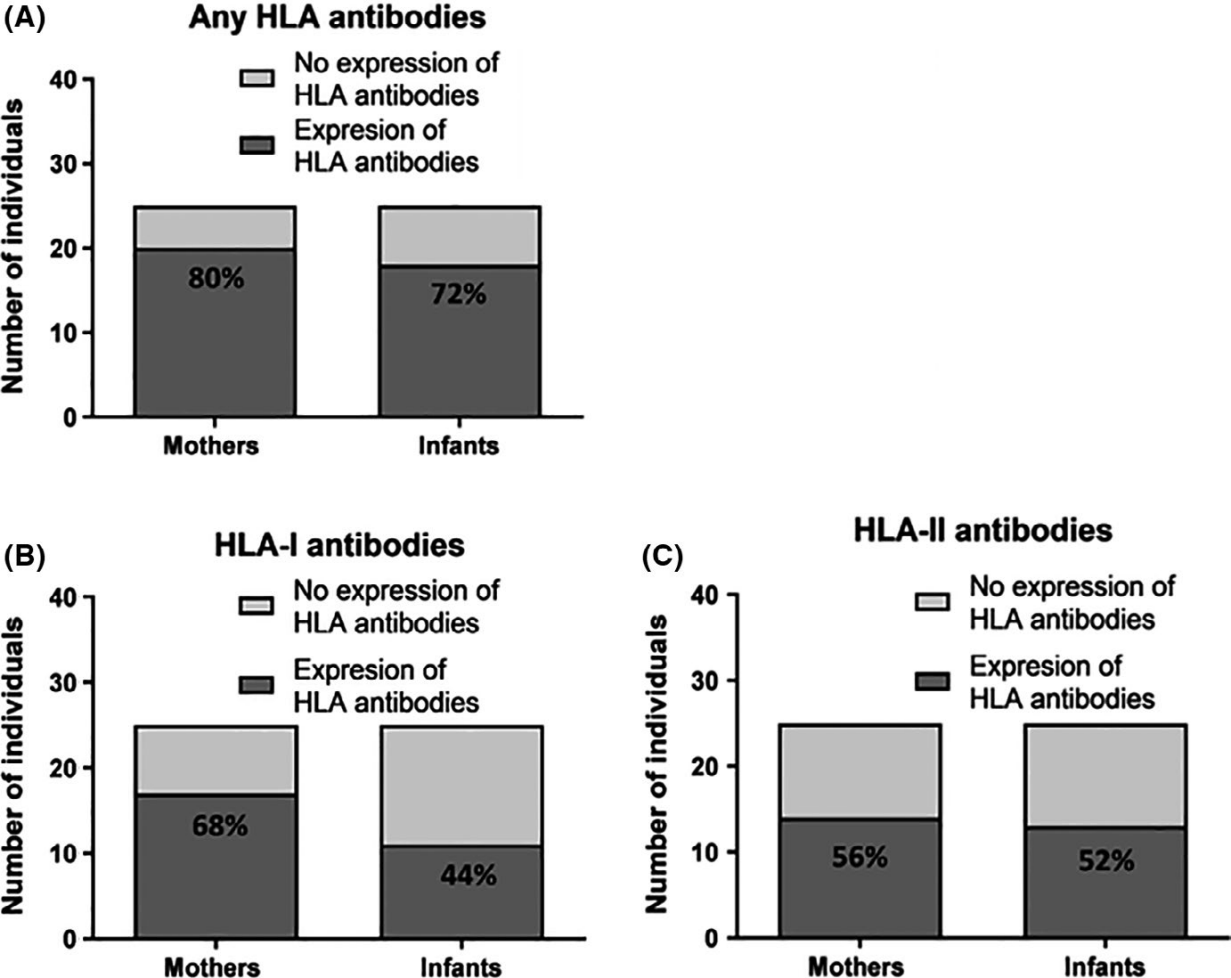
Number (and percentage) of mothers six weeks post‐partum and their six‐week‐old infants, who express HLA antibodies of at least one class (A), HLA class I antibodies (B), and HLA class II antibodies (C) out of total number of individuals (n = 25; all antibodies are IgG)

We first characterized the prevalence of IgG HLA antibodies in mothers and infants. HLA antibodies detected in each mother‐infant pair are listed in Supplementary Tables S1 (class I) and S2 (class II), including those detected only in mother, those detected only in infant, and those that were shared between the two. Our data reveals great diversity in number and type of specificities between the pairs. We found that of 25 mothers, 20 (80%) expressed HLA antibodies of at least one class (Figure 1A), 17 (68%) expressed HLA class I antibodies (Figure 1B) and 14 (56%) expressed HLA class II antibodies (Figure 1C). In infants, of 25, 18 (72%) expressed HLA antibodies of either class, 11 (44%) expressed HLA class I antibodies, and 13 (52%) expressed HLA class II antibodies (Figure 1A, B and C, respectively).

#### 3.1.2 HLA antibody subclasses

**Figure 2:**
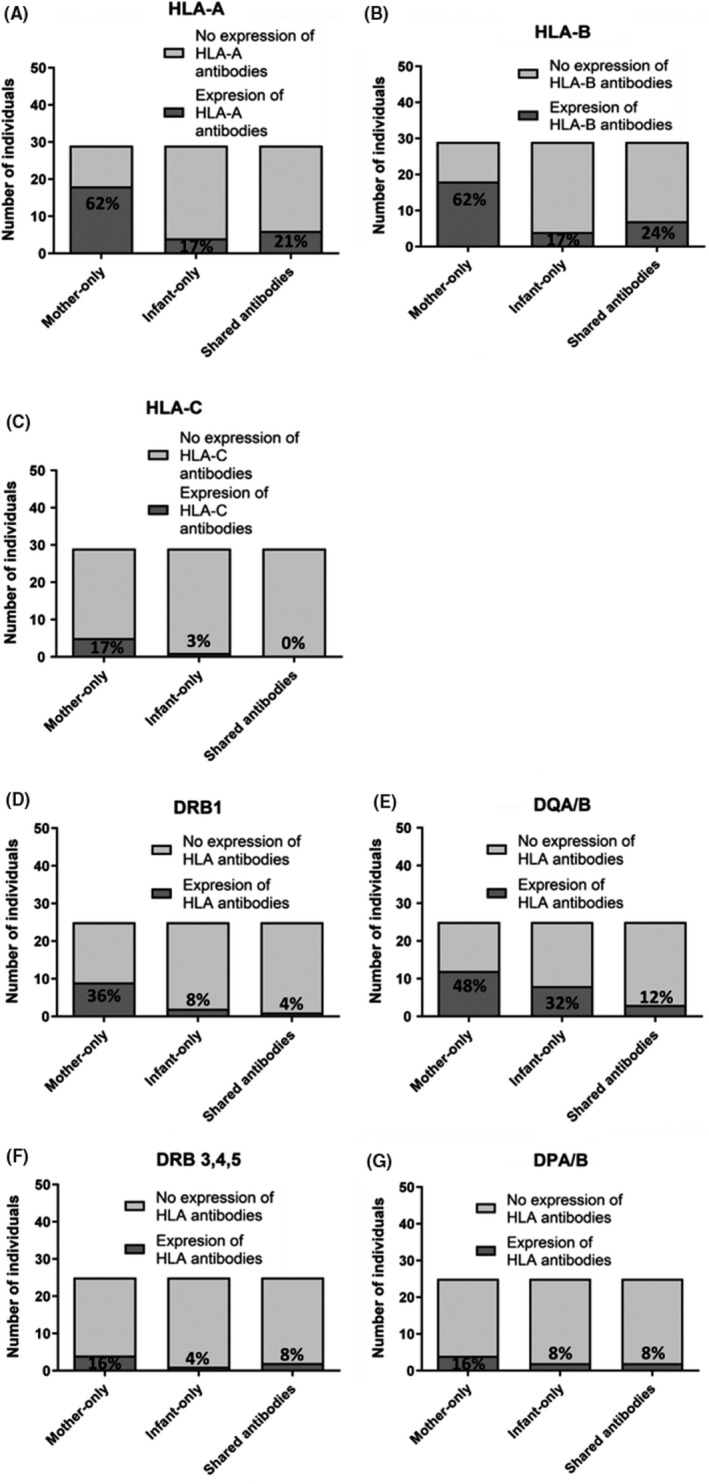
Number (and percentage) of mothers six weeks post‐partum and their six‐week‐old infants, who express HLA class I antibodies (A, B, C for HLA‐A, HLA‐B and HLA‐C, respectively; n = 29) and HLA class II antibodies (D, E, F, G for DRB1, DQA/B, DRB3,4,5 and DPA/B, respectively; n = 25) that were detected only in mothers (Mother‐only antibodies), only in infants (Infant‐only antibodies) and in both mother in infant (Shared antibodies); all antibodies are IgG

Each HLA‐I antibody subclass, namely HLA‐A, HLA‐B and HLA‐C, showed a similar pattern, with a higher proportion of specificities apparent in the mother only, and a low proportion of specificities apparent in infants only and in the group shared between mothers and infants (Figure 2A‐C). More specifically, out of 29 mothers, 18 (62%), 18 (62%) and five (17%) expressed HLA‐A, HLA‐B and HLA‐C antibodies that were not detected in their infants, respectively. However, only five (17%) infants expressed anti‐HLA‐A antibodies, five (17%) expressed anti‐HLA‐B, and only one (3%), expressed anti‐HLA‐C. Similarly, low proportions of antibodies were shared between mothers and infants, with six pairs (21%) expressing HLA‐A antibodies and seven pairs (24%) expressing HLA‐B antibodies; notably no HLA‐C antibodies were shared between mothers and infants.

Similarly, for HLA class II, a high proportion of mothers (9/25, 12/25, 4/25 and 4/25, which represented 36%, 48%, 16% and 16% out of all mothers) expressed DRB1, DQA/B, DRB3,4,5 and DPA/B antibodies, respectively, that were not detected in their infants. There were few infant‐only antibodies (2/25, 8/25, 1/25 and 2/25, equal to 8%, 32%, 4% and 8%, respectively) as well as few shared antibodies (1/25, 3/25, 2/25 and 2/25, equal to 4%, 12%, 8% and 8%, respectively). DQA/B antibodies were the predominant HLA‐II antibody sub‐class in mothers and babies (Figure 2D‐G).

### 3.2 Number of target HLA specificities per individual

**Figure 3:**
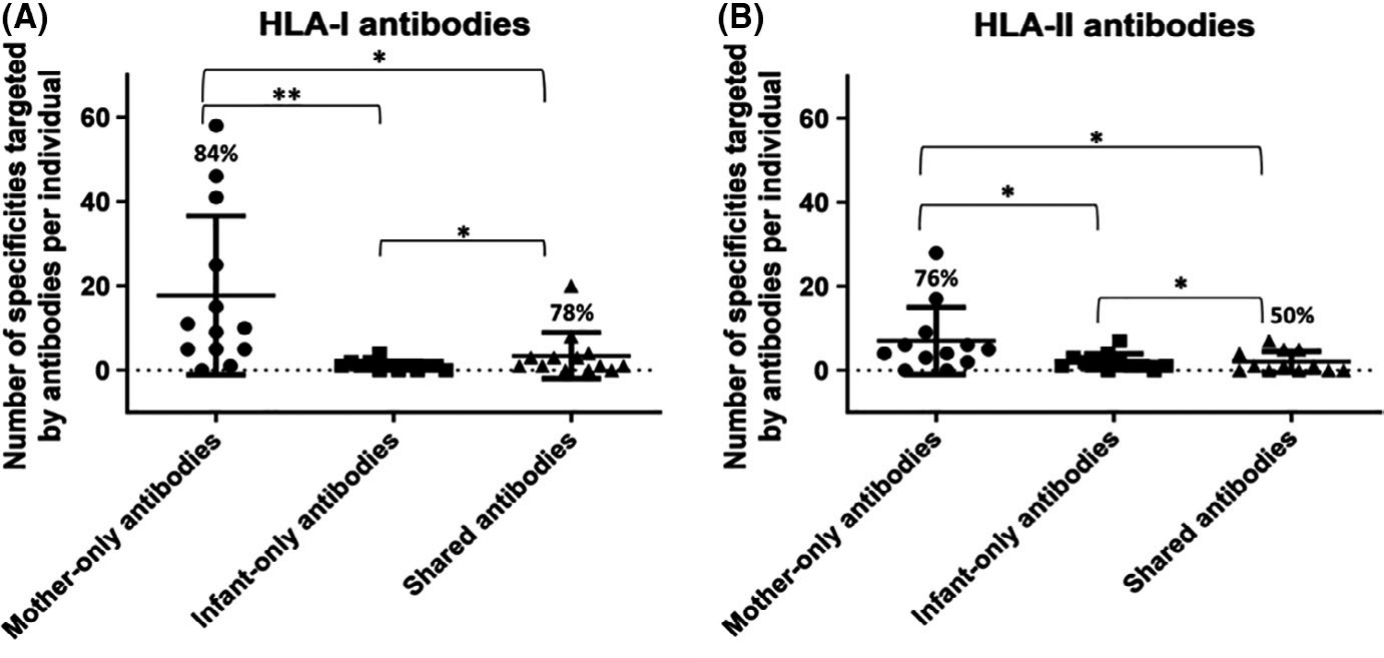
Number of different specificities targeted by HLA antibodies (class I, A and class II, B; all antibodies are IgG) in mothers six weeks post‐partum and their six‐week‐old infants. Number of antibody specificities detected only in mother (Mother‐only antibodies), only in infant (Infant‐only antibodies), or those detected in both mother and infant (Shared antibodies) are shown (n = 29; * represents .05 > *P* value > .001; ** represents a *P* value < .001; *P* value was determined by Student's *t* test; median, 25th and 75th percentiles are shown). On top of the Mother‐only antibodies column—percentage out of total antibodies detected in mothers. On top of the Shared antibodies column—percentage out of total antibodies detected in infants

Next, we measured the number of different HLA specificities targeted by mothers and infants (Figure 3). We sought to determine whether infants expressed the same repertoire of HLA antibodies as their mothers, with the assumption that such antibodies would be of maternal origin. In contrast, we postulated that antibodies detected in infants but not in their mothers were either self‐made, or, less likely—of maternal origin but undetectable in the mothers; and that antibodies detected in mothers but not in their infants were either produced during pregnancy yet not transferred to the foetus, or produced post‐partum.

We found that overall, infants targeted significantly fewer HLA specificities than mothers (*P* value <.05 for both HLA classes). Interestingly, only some of these specificities were shared with their mothers (a mean of 78% for HLA‐I and 50% for HLA‐II, Figure 3A and 3B, respectively). Surprisingly, we also found that most specificities detected in mothers were not detected in their babies (a mean of 84% for HLA‐I and 76% for HLA‐II, Figure 3A and 3B, accordingly).

### 3.3 Expression intensity of HLA antibodies

**Figure 4:**
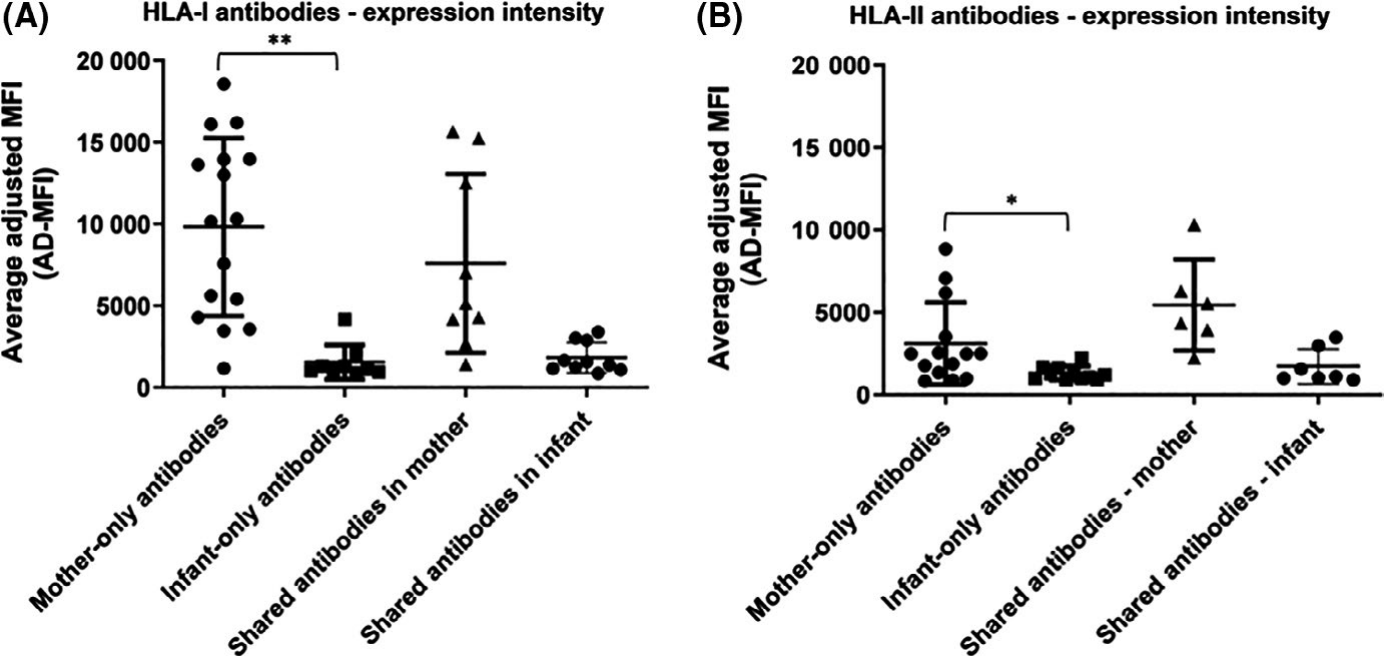
Intensity of antibody expression. Mean AD‐MFI (adjusted MFI as calculated by the MatchIt analysis software) of antibodies detected only in mothers (Mother‐only), only in infants (Infant‐only), and those detected in both mother and infant (Shared antibodies) are shown for HLA‐I (A, n = 17; ** represents *P* value < 0.001) and HLA‐II (B, n = 15; * represents 0.05 > *P* value > 0.001) antibodies (all antibodies are IgG; *P* value was determined by Mann‐Whitney test; median, 25th and 75th percentiles are shown)

The expression intensity was significantly lower for antibodies detected in infants compared with antibodies detected in mothers (p value < 0.001 and < 0.05, presented in Figure 4A and 4B, for HLA‐I and HLA‐II antibodies, respectively). Interestingly, this included antibodies shared between mother and infant, possibly indicating that only a fraction of the amount of antibody produced in mothers crossed the placenta.

### 3.4 Complement‐fixing ability of detected HLA antibodies

**Figure 5:**
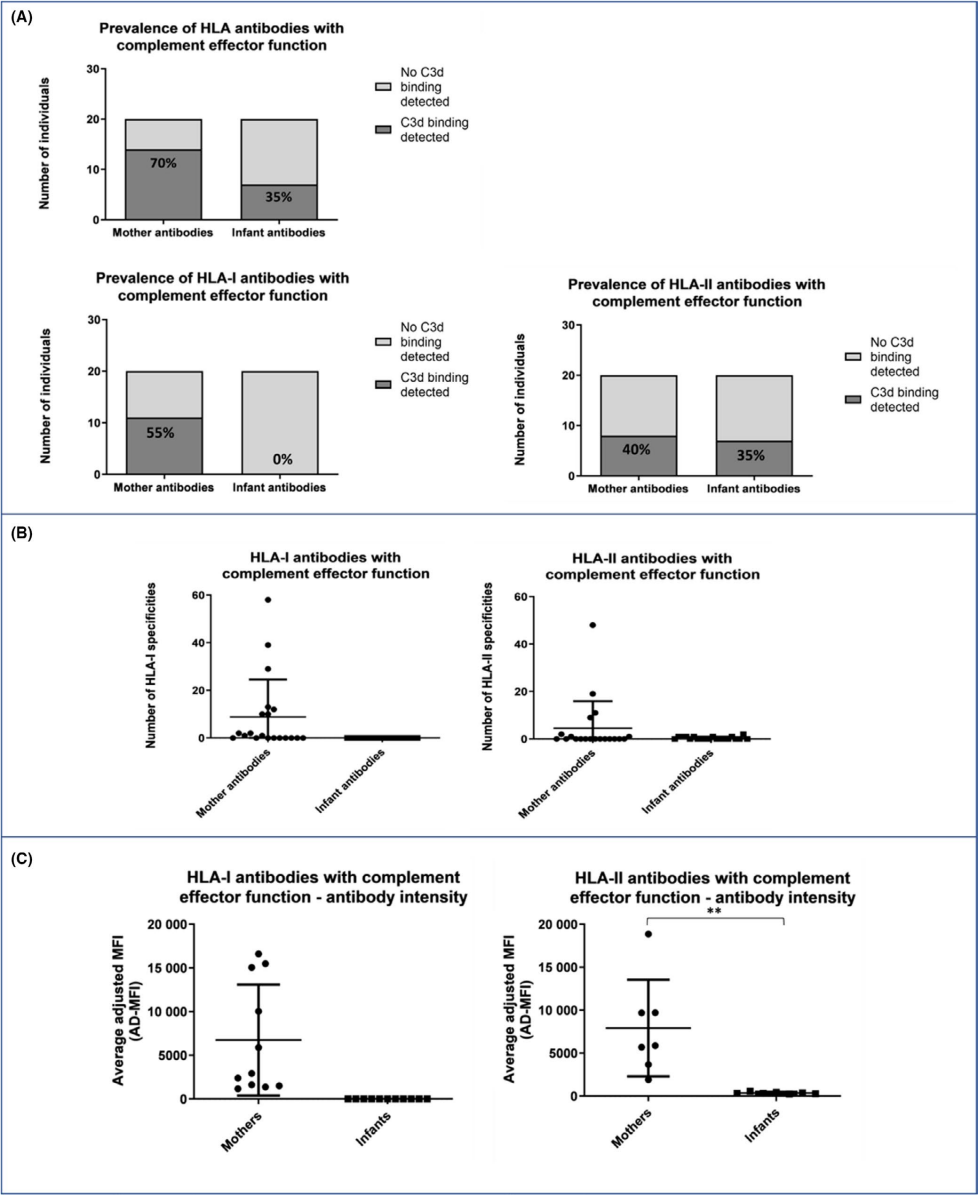
HLA antibodies with complement effector function (as detected by C3d binding) in mothers six weeks post‐partum and their six‐week‐old infants: A. Number (and percentage) of mothers and infants who express complement‐activating HLA antibodies out of total number of individuals (n = 20); B. Number of different HLA specificities per individual of complement‐activating antibodies (n = 20; median, 25th and 75th percentiles are shown); C. Antibody expression intensity (average AD‐MFI as calculated by the MatchIt analysis software) of complement‐activating HLA antibodies (n = 20; ** represents *P* value < .001; *P* value was calculated using Mann‐Whitney test; median, 25th and 75th percentiles are shown)

As expected, we detected complement‐fixing HLA antibodies in mothers, with 55% and 40% expressing HLA‐I and HLA‐II antibodies, respectively (Figure 5A). The number of HLA specificities varied between mothers (Figure 5B) but was in the same range as the maternal HLA IgG antibodies (Figure 3). For HLA‐I, the expression intensity of these complement‐fixing antibodies was generally lower than that observed for maternal IgG; however, for HLA‐II the intensity was overall higher than that of maternal IgG (Figure 5C). In infants, we could not detect complement‐fixing HLA class I antibodies (Figure 5A), indicating that whether self‐made or of maternal origin, HLA class I antibodies do not activate complement at six weeks of age. In contrast, we did detect complement‐fixing HLA class II antibodies in 35 per cent of the infants (Figure 5A). However, HLA class II antibody numbers per individual (Figure 5B) and expression levels (Figure 5C) were extremely low (*P* value <.001 for mean antibody intensity of mothers versus infants), possibly because their production might only begin at six weeks of age. We detected infant complement‐fixing HLA‐II antibodies shared with the mother in only one infant (not shown).

### 3.5 Pregnancy study

**Figure 6:**
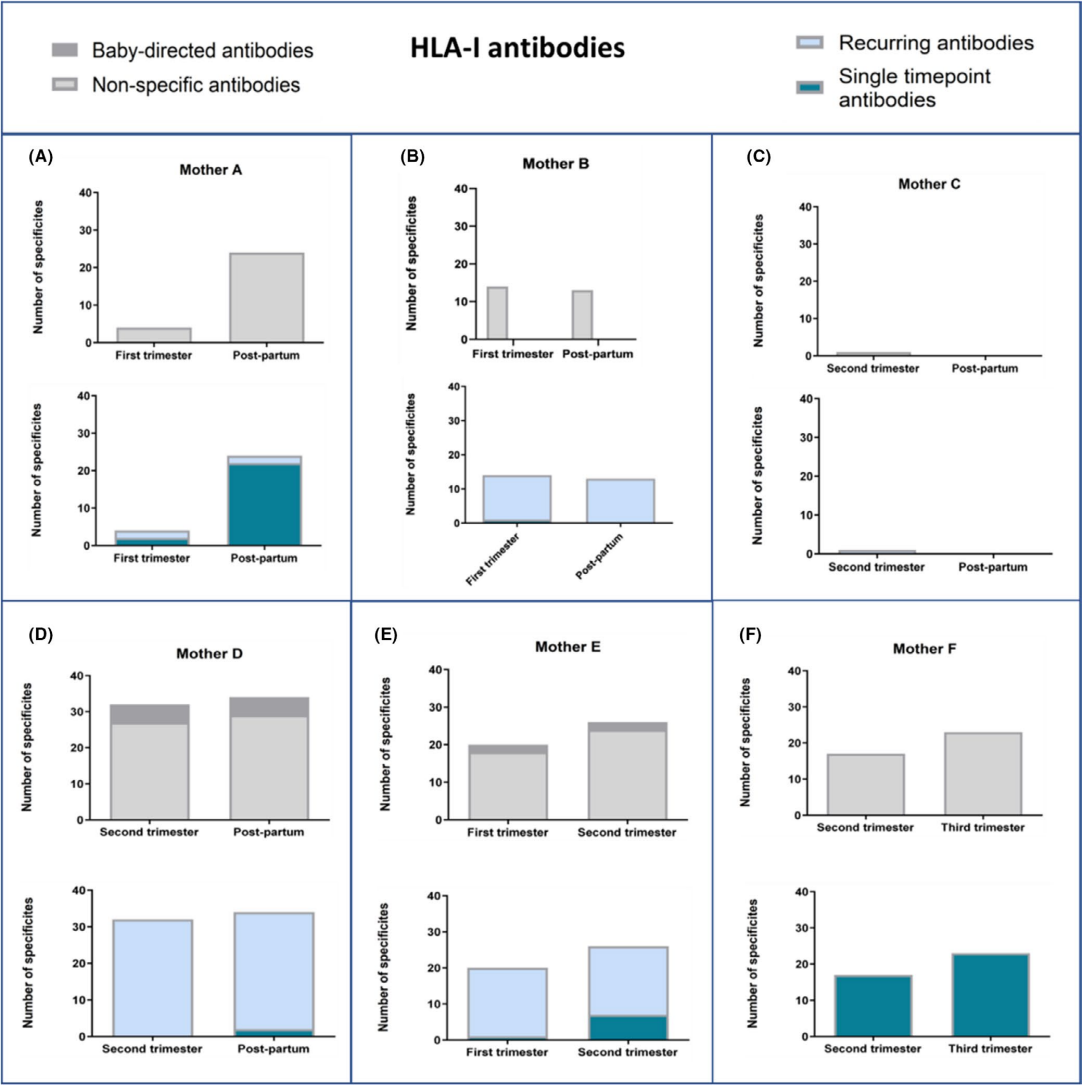
Number of different HLA class I antibodies in six pregnant women (A‐F) as detected at different time points in pregnancy (all antibodies are IgG). Two graphs are shown for each woman: the top graph presents baby‐directed and non‐specific antibodies (shown in dark and pale grey, respectively); the lower graph presents recurring and single time point antibodies (shown in pale and dark blue, respectively). Baby‐directed HLA‐I antibodies were detected in two out of six mothers (mothers D and E). Development of new HLA‐I antibodies is apparent in mothers A, D, E and F. Certain antibody specificities in mothers A, C and F were detected once but not subsequently

**Figure 7:**
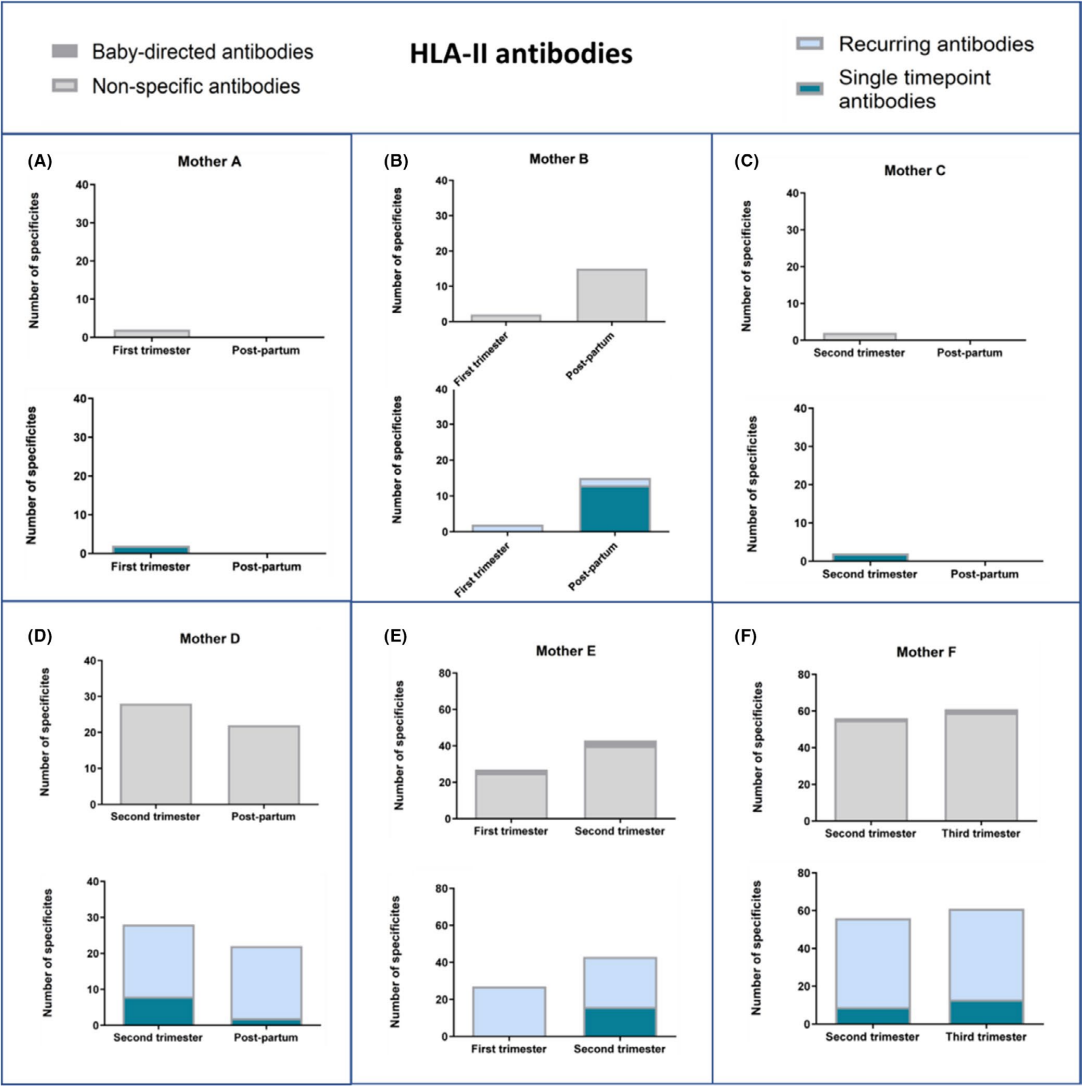
Number of different HLA class II antibodies in six pregnant women (A‐F) as detected at different time points in pregnancy (all antibodies are IgG). Two graphs are shown for each woman: the top graph presents baby‐directed and non‐specific antibodies (shown in dark and pale grey, respectively); the lower graph presents recurring and single time point antibodies (shown in pale and dark blue, respectively). Baby‐directed HLA‐II antibodies were detected in two out of six mothers (mothers E and F). Development of new HLA‐II antibodies is apparent in mothers B, E and F. Certain antibody specificities in mothers A, C, D and F were detected once but not subsequently

In six women, referred to as Mother A‐F, we measured the number and specificities targeted by maternal IgG HLA antibodies (Figure 6, class I and Figure 7, class II) at different stages of gestation and/or post‐partum. All women expressed HLA class I (Figure 6), as well as HLA class II (Figure 7) antibodies in the pregnancy time points tested; however, there were noticeable variations between women in number of specificities. In the post‐partum time point, three out of four women expressed HLA class I antibodies (Figure 6) and two out of four women expressed HLA class II antibodies (Figure 7). We further assessed whether maternal antibodies targeted HLA specificities of the infant by genotyping the infant HLA‐B, HLA‐C, DRB1, DQA/B, DRB3,4,5, DPA/B, and classifying the maternal antibodies into baby‐directed or non‐specific. For both classes, two out of six women (mothers D and E for HLA‐I, and E and F for HLA‐II) expressed baby‐directed HLA antibodies.

Next, we classified the maternal antibodies into single time point (detected only at one time point) or recurrent (detected in at least two time points), in an attempt to assess the appearance of new antibodies. Overall, once antibodies were present in the mother, they recurred at subsequent time points. However, we did find antibody specificities that were detected once but not subsequently, as seen in mothers A, C and F (Figure 6) for HLA‐I and mothers A, C, D and F (Figure 7) for HLA‐II. Development of new HLA antibodies in the second time point compared with the first, was apparent in four mothers for HLA‐I (A, D, E and F, Figure 6), and four mothers for HLA‐II (B, D, E and F, Figure 7).

## 4 DISCUSSION

Currently, it is not known whether HLA antibodies play a role in pregnant women or if they are essential for maintaining a healthy pregnancy. However, many studies suggest that high levels of HLA antibodies, at least (and perhaps only) in late stages of pregnancy, are important for a successful outcome. We found that most women in our two cohorts expressed HLA antibodies in their pregnancy and/or post‐partum (Figures 1, 6, 7). To our surprise, in the mother‐infant cohort, a relatively small fraction of IgG HLA antibodies detected in mothers post‐partum was found in the infants (these were termed “shared antibodies”). If we assume that the mothers produced the HLA antibodies detected post‐partum during pregnancy, the low levels of shared antibodies in infants suggests selection in transplacental crossing. So far, the efficiency of placental crossing has not been shown to depend on antibody specificity, and hence, the mechanism behind such selection remains to be explored.

It is also possible, however, that pregnant women produce and transfer HLA antibodies to the foetus, but rather than being selected against at placental crossing, some of the transferred antibodies might be degraded in the infants by the time they are six weeks of age, thus resulting in lower prevalence and intensity of shared antibodies in infants compared with mothers.

Alternatively, some of the maternal HLA antibodies we detect in mothers post‐partum may have been produced during the first six weeks after delivery rather than during pregnancy. Theoretically, HLA antibodies produced post‐partum may still be passed on to the infants through breastfeeding. However, this would likely be at low levels as transfer of IgG antibodies via breastmilk is limited.^55^, ^56^

In the future, it would be interesting to explore the possibility of selection in transplacental crossing of HLA antibodies, including possible post‐translational modifications that would increase their FcRn binding affinity, and a potential link between such modifications and antigen specificity. One interesting aspect of a theoretical antibody selection would be the fate of antibodies that may become autoantibodies in the foetus, directed at paternal HLA proteins. In our longitudinal pregnancy study, half of the women expressed a small proportion of baby‐directed HLA antibodies of at least one class. However, we do not know whether these antibodies were transferred to the foetuses, and if so, what impact they might have had on the foetal and later, infant immune system.

Another interesting aspect of a potential selection is the transfer of the HLA‐C antibodies. In the mother‐infant cohort, we found that the shared antibodies did not include any HLA‐C antibodies, which may be due to our small sample size, especially in light of the small proportion of maternal HLA‐C antibodies within the class‐I HLA antibodies. However, it is also possible that HLA‐C antibodies are less preferentially transferred to the foetus. Interestingly, at the beginning of pregnancy, placental trophoblast cells express HLA‐C proteins on their membranes, later internalizing them. The levels of HLA‐C in placental trophoblast cells are then highly increased at birth.^57^ Hypothetically, HLA‐C antibodies may be selected against for transplacental crossing, possibly to lower the chances of transferring autoantibodies to the foetus. However, at this point, this scenario remains speculative.

Unlike antibodies targeting pathogenic antigens, whose role is well understood, the role of HLA antibodies in the foetus, and later in the child, is not known. A study by Gros and colleagues^58^ suggested that maternal antibodies transferred to the foetus during pregnancy or to the infant via breast milk may act as immunomodulators, leading to the development of specific and long‐lasting immune responses in the infant. However, whether this is the case for HLA antibodies is not known. It has been shown that antibodies against a variety of HLA alleles may be produced after exposure to a specific HLA antigen.^59^ The significance of HLA alloimmunity and whether humans need to develop immunological tolerance or alloimmunity against HLA proteins, remain to be established.

HLA antibodies may play a crucial role in infection with enveloped viruses, such as the Human Immunodeficiency Virus (HIV), Epstein‐Barr Virus (EBV), influenza or measles. While budding out of host cells, enveloped viruses incorporate different host proteins, including HLA, which they then introduce to new hosts.^60^ Therefore, individuals who express HLA antibodies may have pre‐existing immunity to pathogens. However, whether this is beneficial, harmful or simply irrelevant, still needs to be determined. If pre‐existing antibodies increase protection, we expect that the more HLA antibodies present, the higher their protection. However, it is also possible that HLA antibodies may, in fact, increase susceptibility to certain viruses. This may be due to the HLA antibody‐induced recruitment of immune cells that some viruses may easily infect. To note, previous studies in macaques and non‐human primates have suggested a protective role for HLA antibodies against infection with the enveloped simian immunodeficiency virus (SIV).^61^, ^62^, ^63^ Interestingly, it was found that administration of vaccines against infectious agents such as the influenza and measles viruses may induce de‐novo production of HLA antibodies^64^, ^65^, ^66^; the impact of these antibodies on future infections remains to be determined. It is tempting to envision the use of HLA antibody‐based alloimmunity for vaccine strategies in pathogenic diseases caused by enveloped viruses that incorporate host‐derived HLA proteins or those like HIV that express viral proteins with high structural homology to HLA proteins.^62^, ^67^, ^68^, ^69^, ^70^ Interestingly, antibody transfer from mother to foetus is compromised in various conditions affecting placental integrity, including placental malaria and HIV infection.^15^, ^16^, ^30^, ^71^, ^72^ It would be interesting to see whether these conditions also impact the transfer of HLA antibodies, and if so, what effect impaired transfer has on susceptibility to infection in the foetus and after birth.

It has recently been shown that in the first weeks of life, the infant's immune system undergoes drastic changes that comprise maturation of most immune cells, including B lymphocytes.^73^, ^74^ This rapid maturation results in differences between the immune profile at birth (as seen in cord blood) and that of infants several weeks of age. Maturation may include the ability of B cells to switch from IgM (which is already produced in the foetus) to other types of antibodies, presumably in response to specific antigens. In our study, we find that at six weeks of age, infants express IgG HLA antibodies, including a considerable number of antibodies that are not found in their mothers, which may be self‐made antibodies. Whether these antibodies are produced as “natural antibodies” or in response to specific antigens warrants further investigation. To note, we cannot exclude the possibility that at least some of the HLA antibodies found in infants but not in their mothers, are in fact of maternal origin, having been present antenatally yet becoming undetectable in the mother post‐partum.

In our study, we show that amongst HLA antibodies produced by the mothers, some antibodies have complement effector functions, likely in keeping with IgM isotype. The complement system may be activated by IgM or IgG antibodies; however, unlike IgG, which has multiple effector functions, complement activation is almost the sole effector function of IgM antibodies.^1^ Thus, in infants, the absence of complement‐fixing HLA class I (but not class II) antibodies suggests that their HLA‐I antibodies are unlikely to be IgM.

### 4.1 Limitations and strengths

Strengths of this study include the use of highly sensitive single‐antigen technology which can detect low intensity antibodies. The study is unusual in that it includes healthy Black African women and infants with no underlying infectious diseases. Additionally, we explored multiple HLA loci including class II loci, rarely analysed in previous studies. A main limitation of this study is that we could not test any of the women prior to their pregnancy due to lack of available samples, and therefore our findings in pregnancy and post‐partum could not be compared with a basal, non‐pregnant state. In the mother‐infant cohort, we did not have cord blood samples available, and therefore could not compare the infant samples at six weeks to those at birth. Additionally, we had no DNA available to explore the presence of antibodies directed at the infants’ HLA. In the pregnancy cohort, we had HLA type of the infants but there was no infant serum available to establish whether baby‐directed antibodies crossed to the baby. Additionally, we did not HLA type the mothers so could not confirm whether antibodies were anti‐paternal. Gravidity and parity data were not collected in the mother‐infant study, and thus, the possible effect of these on HLA antibody repertoire could not be tested.

### 4.2 Summary

In this study, we show that up to 80 per cent of women after birth express IgG HLA antibodies. Surprisingly, we detect less than 25 per cent of these antibodies (16 per cent for HLA‐I and 24 per cent for HLA‐II) in the neonate, raising the possibility of HLA antibody selection in placental transfer. Our study also suggests that infants start producing their own IgG HLA antibodies in the first weeks of life. Further investigation is required to confirm these two hypotheses and the mechanisms behind them. The presence of HLA antibodies in neonates may impact their immune reaction to HLA proteins, including those incorporated in enveloped viruses. Whether and how HLA antibodies affect susceptibility to enveloped viruses needs to be further explored using prospective studies. Overall, our findings may provide a preliminary baseline for future studies focusing on pregnancy complications that are not fully understood yet, such as preeclampsia, as well as infections with (and possibly vaccines against) enveloped viruses in new‐borns, in which HLA antibodies may play a role.

## CONFLICT OF INTEREST

The authors declare no conflict of interests.

## Supporting information

Table S1‐S2
