# Supplementary material for: HLA antibody repertoire in infants suggests selectivity in transplacental crossing
Source: Am J Reprod Immunol. 2020 Jun 16;84(2):e13264. doi: 10.1111/aji.13264 (PMC7507134; doi:10.1111/aji.13264)
Supplement: Supplementary file 1 — Table S1‐S2 [file AJI-84-e13264-s001.docx]

**Supplementary Table 1**: List of specificities targeted by HLA class I antibodies in mothers-only antibodies, infants-only antibodies and antibodies shared by mothers and their infants (six weeks after birth); hyphen – no antibodies detected.

**Supplementary Table 2**: List of specificities targeted by HLA class II antibodies in mothers-only antibodies, infants-only antibodies and antibodies shared by mothers and their infants (six weeks after birth); hyphen – no antibodies detected; n/a – not analysed.
